# Independent Discovery of *Mesomycoplasma conjunctivae* in Non-Native Aoudad (*Ammotragus lervia*) in Texas

**DOI:** 10.3390/ani16172639

**Published:** 2026-08-23

**Authors:** Emily A. Wright, Brendan R. Amman, W. Indiana Beck, Caleb D. Phillips, Robert D. Bradley

**Affiliations:** 1 Independent Researcher, Rockwall, TX 75032, USA; 2 Department of Biological Sciences, Texas Tech University, 2901 Main St., Lubbock, TX 79409, USA; bamman@ttu.edu (B.R.A.); caleb.phillips@ttu.edu (C.D.P.); robert.bradley@ttu.edu (R.D.B.); 3 Independent Researcher, Needham, MA 02494, USA; indy.w.beck@gmail.com; 4 Natural Science Research Laboratory, Museum at Texas Tech University, 3301 4th St., Lubbock, TX 79409, USA

**Keywords:** 16S rRNA sequencing, aoudad, bighorn sheep, conservation, disease surveillance, haplotype network, *Mesomycoplasma*

## Abstract

Conservation of native species is an ongoing task, especially when non-native species present a threat via competition and disease transmission. In Texas, native desert bighorn sheep (DBS) compete directly with non-native aoudad for resources. Aoudad (*Ammotragus lervia*, also known as Barbary sheep), introduced into the United States from Africa, are competent hosts of *Mesomycoplasma ovipneumoniae* and are thought to be the primary reservoir for transmitting pneumonia to DBS. A recent epidemic (circa December 2025) of infectious keratoconjunctivitis (IKC) in aoudad was reported in western Texas. Herein, we compare newly derived microbial data (16S rRNA sequencing) to results derived from a previous dataset (multi-locus sequence typing). Five *M. conjunctivae* haplotypes were indicated by 16S sequence data obtained from 33 individuals. Phylogenetic analyses identified these haplotypes as members of a clade containing sequences from wild ungulate hosts, whereas sequences from domestic ungulates formed a divergent clade (6.58%). Together, these data indicate the presence of an aoudad-like *M. conjunctivae* obtained from four mountain ranges in western Texas during 2018–2020. These implications suggest that monitoring for IKC and identifying the bacterial pathogen is paramount in case of spillover from aoudad to DBS. Based on the data presented herein, effective management strategies should take a dual approach of bacterial surveillance and preparation in case of antibiotic intervention for DBS conservation.

## Simple Summary

A continual outbreak of infectious keratoconjunctivitis (IKC) in non-native, free-ranging populations of aoudad (*Ammotragus lervia*) in western Texas has been reported since December 2025. It seemed prudent to revisit samples obtained from the last 6–8 years to identify if this is truly a new or an undetected bacterial pathogen that has been circulating for some time. Herein, we combined a newly generated microbiome dataset with previous findings using 16S rRNA sequencing. Five haplotypes of *Mesomycoplasma conjunctivae* were identified using an alignment of 406 bp in aoudad populations in the Carrizo, Chinati, Sierra Vieja, and Van Horn mountains between 2018 and 2020. The aoudad-like *M. conjunctivae* is most genetically similar to a strain of *M. conjunctivae* sourced from a moose. Interestingly, both wildlife strains of *M. conjunctivae* grouped in a clade sister to strains of *M. conjunctivae* from domestic goats, suggesting that there may be a phylogenetic relationship between the bacteria and host and divergence between wildlife and livestock strains of *M. conjunctivae*. Because aoudad live in sympatry with native desert bighorn sheep (DBS), a highly prioritized conservation management species, it is imperative to opportunistically sample IKC-affected aoudad to determine the bacterial cause of disease and prepare a plan for medicinal intervention if IKC is transmitted into DBS populations.

## 1. Introduction

When considering North American megafauna, most, if not all, have required human intervention to prevent extirpation or extinction [1,2,3]. Once management tools were in place, such as translocation efforts, restoration of habitat, construction of year-round water sources, reduction in livestock competition, hunting restrictions (seasons and bag limits), and other means, most species became self-sustaining, needing little to no human assistance. Bighorn sheep (*Ovis canadensis*) range-wide are an exception, in which significant efforts and resources have been necessary to maintain populations [4], especially in the Chihuahuan Desert of western Texas, where the desert bighorn sheep (DBS) have required substantial human intervention to persist [5,6].

DBS in Texas were extirpated in the early 1960s as a response to the constant pressure of competition and disease transmission with domestic sheep and goats, predation, and overharvesting [5,6,7]. TPWD and conservation organizations combined efforts to restore and translocate DBS across their historical mountain ranges in Texas. These efforts have been met with mixed results. For example, periodic bluetongue (*Orbivirus caerulinguae*, [8]) epizootic events have curtailed population expansion [7]. Recently, a new transmission pathway stems from aoudad (*Ammotragus lervia*), an introduced and invasive montane ungulate from northern Africa [9,10,11]. Known to harbor *M. ovipneumoniae* asymptomatically [12,13,14,15], aoudad populations have grown exponentially since their introduction in the 1930s [16,17], compete for resources [18], and now occur in several ecoregions, including the Trans-Pecos, Edwards Plateau, and Panhandle (High and Rolling Plains).

Recent observations of aoudad mortalities by landowners, outfitters, and hunters have been confirmed by the Texas Parks and Wildlife Department (TPWD) as infectious keratoconjunctivitis (IKC), otherwise known as pinkeye. This current IKC epidemic has been observed in Brewster, Jeff Davis, and Presidio counties with aoudad showing severe clinical characteristics (e.g., blindness) in one or both eyes [19]. TPWD believes that *Moraxella* spp. are causing this outbreak [19]. However, other bacterial species, such as *Mesomycoplasma conjunctivae*, can cause IKC. For example, in Arizona, domestic goats transmitted IKC to DBS, in which *M. conjunctivae* was identified as the culprit [20,21]. Other studies involving muskoxen (a sheep/goat relative) identified *M. conjunctivae* as the bacterial pathogen during an IKC outbreak in Norway [22].

Interestingly, in addition to documenting the presence of *M. ovipneumoniae* in DBS and aoudad, [14] discovered potentially two additional *Mesomycoplasma* spp. in the nasal cavity of aoudad individuals. At the time, [14] proposed that these bacteria were most likely aoudad-like strains representative of *M. conjunctivae* and *M. hyopneumoniae* and were either (1) real and novel to Caprinae, (2) a product of contamination, or (3) misidentified based on partial sequences of 16S and IGS queried into NCBI Blast.

With the current outbreak of IKC in aoudad, our goal was to establish a temporal perspective concerning the presence of IKC-causing bacteria from previous samples (collected in 2018–2020) stored in a natural history collection. Consequently, we used an external laboratory (MicroGenDx, Lubbock, Texas) to conduct 16S rRNA sequencing from aoudad captured in the Carrizo Mountains, Texas, in combination with the 16S dataset from [14]. The goal was to characterize the nasal and tonsil microbiome relative to identifying causative bacteria associated with IKC.

## 2. Materials and Methods

### 2.1. Sampling

A total of 21 free-ranging aoudad from the Carrizo Mountains in portions of Culberson and Hudspeth counties in western Texas were opportunistically captured in collaboration with TPWD and private landowners. Once animals were restrained using aerial net gun deployment measures from a helicopter, they were delivered to a staging area. Tissues were obtained while monitoring the temperature and overall well-being of each animal. Samples included blood, serum, ear clip, fecal pellets, hair, four nasal swabs, and three tonsil swabs. None of the 21 aoudad presented clinical IKC symptoms during sampling. Individuals were also given unique ear tags as they were part of an inoculation study [23]. All samples collected by Texas Tech University personnel were immediately stored in liquid nitrogen and deposited in the Robert J. Baker Genetic Resources Collection at the Natural Science Research Laboratory, a division of the Museum at Texas Tech University, in perpetuity.

### 2.2. DNA Extraction and 16S rRNA and Sanger Sequencing

Of the 21 individuals, a total of 33 swabs, which included 12/21 aoudad with both nasal and tonsil swabs, 7/21 aoudad with only tonsil swabs, and 2/21 aoudad with only nasal swabs, were sent to MicroGenDX (Lubbock, TX, USA) for DNA extraction, 16S rRNA sequencing, quality control, and bioinformatics. All methods are described in detail following [24].

A subset (n = 12) of these individuals followed [14] for DNA extraction using a subsample of nasal swabs, amplification, and Sanger sequencing of two genes: 16S rRNA and RNA polymerase beta subunit (*rpo*B). All sequences were submitted to NCBI GenBank with accession numbers PZ638879-PZ638894 (16S rRNA) and PZ674026-PZ674030 (*rpo*B).

### 2.3. Phylogenetic Analyses

#### 2.3.1. Raw Sequence Processing and Final Dataset

We used the Convergent Animal + Human (CAH) FastTrack classification pipeline “https://convah.com/manuscriptreview” (accessed on 18 June 2026) to screen paired-end reads from aoudad nasal and tonsil swabs for bacterial taxa associated with infectious keratoconjunctivitis. Raw reads were processed through a Kraken2-based workflow against the standard-16 database, generating taxonomic classifications. We used the extraction tool to isolate reads classified as *Mesomycoplasma* spp. and aligned them to a reference *M. conjunctivae* genome (ASM2676v1) using BWA.

Although the aoudad-like *M. conjunctivae* was detected in all individuals, we only included sequences from individuals that encompassed the entire targeted 316 bp region and possessed more than 1X coverage in any given area (minimum allowance: 8X and maximum: 19,298X), resulting in 14/21 aoudad from the Carrizo Mountains passing quality control. Some sequences possessed both cytosine and thymine at a single nucleotide position, shown in bold (region: 5′–TGGGGGTGCGCAACATTAG**Y**–3′). When these two alleles were present, the predominant nucleotide was called at a minimum depth of 800 using Integrative Genomics Viewer (IGV) for all downstream analyses [25]. At another nucleotide site, 16S rRNA sequencing based on [24] and traditional 16S Sanger sequencing based on [14] produced either guanine or cytosine nucleotide bases (as described above and at region 5′–TTGAACGG-AATAT**S**–3′). When both datasets were available, chromatograms from Sanger sequencing were manually inspected to authenticate variant calls and resolve intragenomic heterogeneity. These verified consensus sequences were used for analyses and submission to NCBI GenBank.

**Figure 1:**
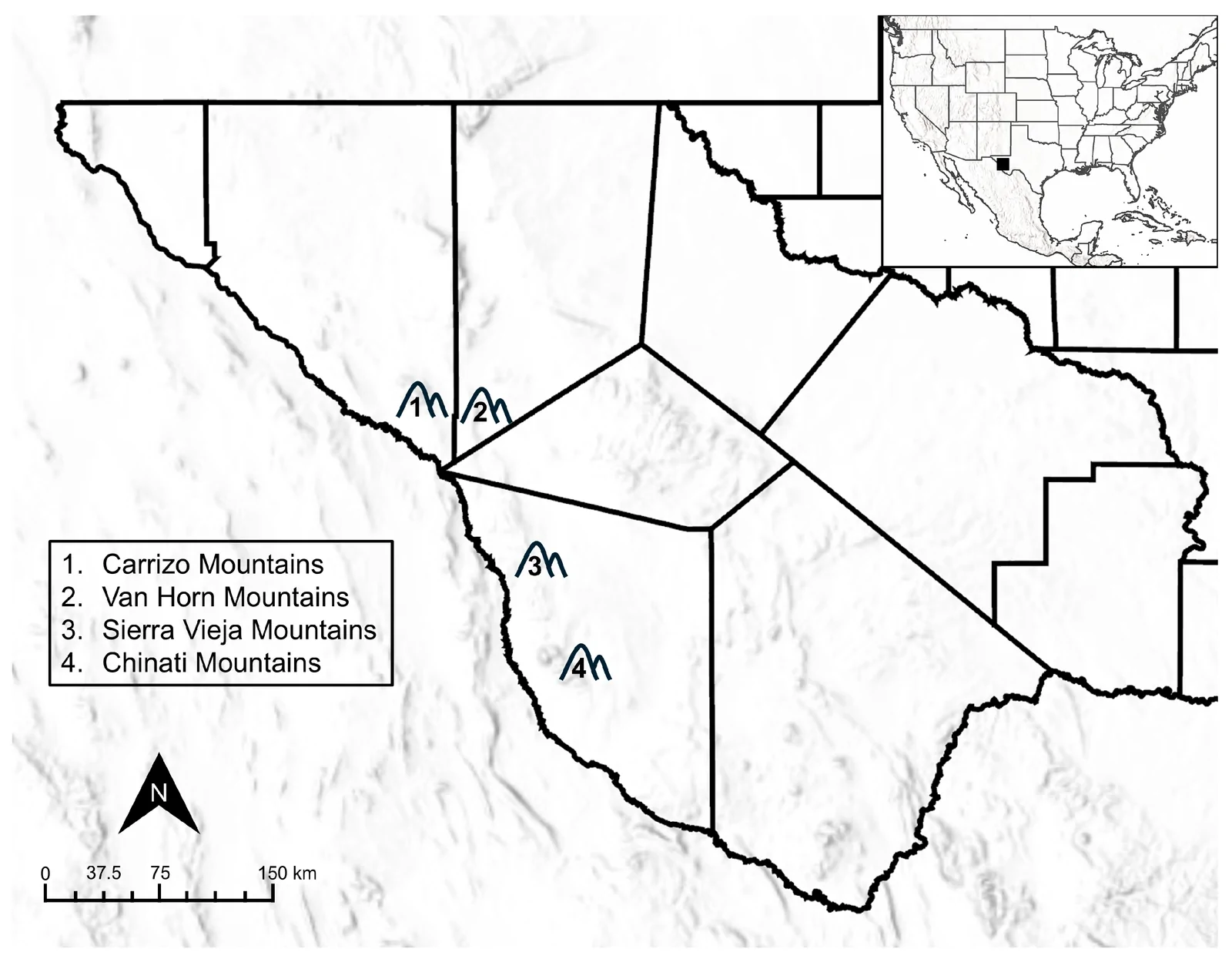
Map of western Texas. Mountain symbols indicate approximate locations for all four mountain ranges, including the Carrizo, Chinati, Sierra Vieja, and Van Horn Mountains.

16S rRNA sequence data (OR552904, OR552905, OR552908, OR552910-OR552918) from [14] were retrieved from NCBI GenBank to compare previously described *M. conjunctivae*-like bacterial strains from 12 free-ranging aoudad in the Chinati, Sierra Vieja, and Van Horn mountains (Figure 1). Further, DNA sequences from the 16S rRNA gene were extracted from six reference genomes, including *M. bovoculi*, *M. conjunctivae*, *M. dispar*, *M. flocculare*, *M. hyopneumoniae*, and *M. ovipneumoniae* (all derived from livestock hosts) as well as from a potential novel *Mesomycoplasma* spp. from a moose [26]. An additional five 16s rRNA sequences of *M. conjunctivae* were obtained from *Capra hircus* (domestic goat) and *Ovis aries* (domestic sheep). Consequently, 406 bp were aligned and included in the final dataset comprising 28 sequences of an aoudad-like *M. conjunctivae* and 12 closely related *Mesomycoplasma* species (see Table S1 for more information).

#### 2.3.2. Phylogeny

To establish genetic associations among individual sequences, a neighbor-joining analysis (PAUP* version 4.0a169) [27], based on genetic distances, was generated using the uncorrected (“p”) model of evolution. *Mycoplasmoides pneumoniae* (genome assembly 50648_A01-3) was designated as the outgroup and *M. neurolyticum* (genome assembly 50648_E01-3) as an ingroup taxon with the final dataset of 38 *Mesomycoplasma* species, totaling 40 sequences. A subsequent bootstrap analysis [28] with 1000 iterations and the “fast” step-wise option was selected to evaluate nodal support.

#### 2.3.3. Diversity and Haplotype Network for *M. conjunctivae*

Genetic distances were estimated using the p-distance model in MEGA12 [29]. The number of polymorphic sites (*s*), nucleotide diversity (π), number of haplotypes (*h*), haplotype diversity (H_d_), and Fu’s test of neutrality were calculated for the final dataset using DNAsp v6 [30]. A haplotype network was generated using PopART [31] and TCS Networks [32] to discern relationships between haplotypes.

#### 2.3.4. Diversity and Haplotype Network for *M. ovipneumoniae*

A haplotype network was generated using PopART [31] and TCS Networks [32] to discern relationships between haplotypes. A known sheep (MN037020) and goat strain (MN037129) of *M. ovipneumoniae* from [33] were used to determine strain type among Texas strains. All published *rpoB* sequences from Texas were retrieved from NCBI GenBank for comparison. The number of polymorphic sites (*s*), nucleotide diversity (π), number of haplotypes (*h*), haplotype diversity (H_d_), and Fu’s test of neutrality were calculated for the final dataset using DNAsp v6 [30]. Genetic distances were estimated using the p-distance model in MEGA12 [29].

### 2.4. Statistical and Community Ecology Analyses

Considering the unequal sampling (33 total swabs from 21 distinct individuals: 12 paired nasal and tonsil swabs, 2 nasal swabs-only, and 7 tonsil swabs-only; see Table S2), sequence count data were zero-filled using the complete function in tidyr to guarantee a fully resolved matrix across all individuals and microbial operational units. All ecological and statistical modeling were executed in R (v4.5.2) utilizing vegan (v2.7-5) and pheatmap packages (1.0.13).

To characterize alpha diversity, the Shannon Diversity Index was calculated for each sample to capture community richness and evenness. Because sample type (nasal and tonsil swab) varied among individuals, we used non-parametric two-sided Wilcoxon Rank-Sum and Kruskal–Wallis tests to compare alpha diversity across tissue types, host sexes, and host age classes. To ensure that host dependency did not bias the tissue comparison, a confirmatory paired Wilcoxon Signed-Rank test was subsequently used strictly on the subset of individuals (n = 12) that had both nasal and tonsil swabs.

Beta diversity was evaluated by calculating a dissimilarity matrix using the Bray–Curtis distance. A multi-factor Permutational Multivariate Analysis of Variance (PERMANOVA) was conducted with the adonis2 function using 999 permutations to characterize microbial community structure. Further, the homogeneity of multivariate dispersions among tissue types was evaluated using the betadisper function, followed by a permutation test to guarantee PERMANOVA assumptions were met. To control for potential confounding host demographics, the model was evaluated using marginal effects (by = “margin”) to simultaneously isolate the variance explained by tissue type, host sex, and host age group. Distinct community structure was demonstrated using Principal Coordinates Analysis (PCoA).

To identify the specific taxonomic drivers characterizing each anatomical niche, a Similarity Percentage (SIMPER) analysis was used to determine the percentage contribution of individual taxa to the overall community dissimilarity between nasal and tonsil environments. A ‘hybrid’ core microbiome list was generated by merging the top 20 most abundant taxa overall with the top 20 most discriminatory taxa isolated by the SIMPER analysis. A final heatmap was generated using Ward’s hierarchical clustering (ward.D2) on a Log_10_-transformed matrix (utilizing a pseudocount of 0.01) to effectively resolve low-abundance, highly discriminatory niche specialists.

## 3. Results

### 3.1. Phylogenetic Analyses

#### 3.1.1. Phylogeny

**Figure 2:**
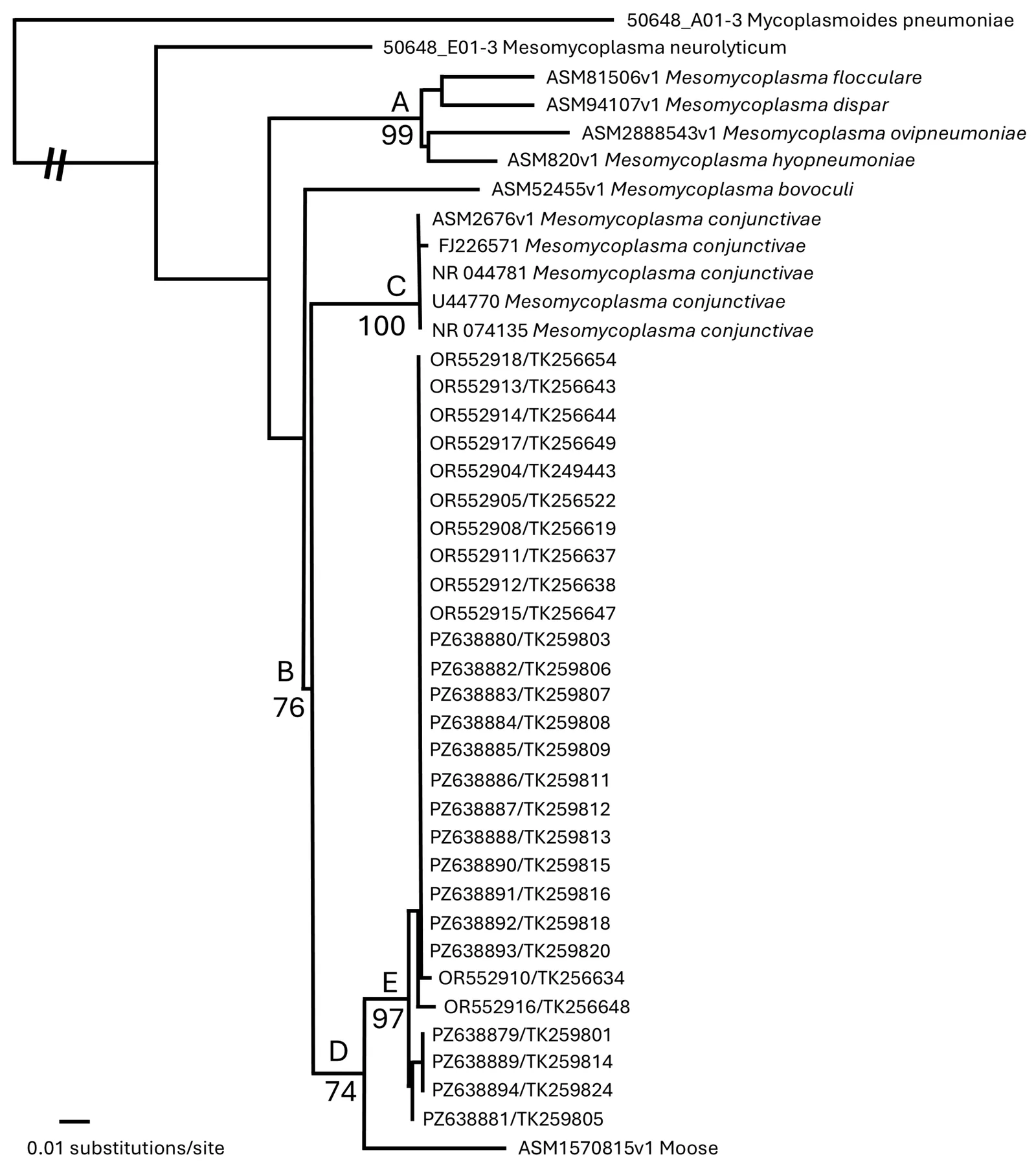
Neighbor-joining phylogeny with the 28 *Mesomycoplasma conjunctivae* sequences derived from nasal swabs of free-ranging aoudad in western Texas. Clade designations are above nodes and bootstrap values are located below nodes.

The neighbor-joining with bootstrap analysis indicated support for five clades (A, B, C, D, and E), with the outgroup *My. pneumoniae* (Figure 2). The first, Clade A, contained the representative sequence for *Me. dispar*, *Me. flocculare*, *Me. hyopneumoniae*, and *Me. ovipneumoniae*. Clade B contained 35 sequences of *Mesomycoplasma* species that were subdivided into Clades C, D, and E. Clade C contained five individuals previously identified as representative of the goat strain of *Me. conjunctivae*. Clade D possessed the potential wildlife strains (i.e., host is a non-domesticated species) of *M. conjunctivae* from one moose and 28 aoudad. Clade E solely contained 28 sequences from aoudad individuals, assignable to a tentatively identified wildlife strain of *Me. conjunctivae* [14]. The association between Clades A and B was unresolved, whereas all other clades are supported by bootstrap values (BS > 65).

#### 3.1.2. Diversity and Haplotype Network for *M. conjunctivae*

The genetic distances among *Mesomycoplasma* species (Table 1), obtained from an aligned 406 bp region, indicate that the aoudad-like *M. conjunctivae* (Clade E) is most genetically similar to a previously described wildlife strain of *M. conjunctivae* from a moose (mean: 5.20%, range: 5.05–6.52%). The goat-like strains of *M. conjunctivae* (Clade C) also were genetically similar to the moose-like *M. conjunctivae* (mean: 8.06%) and aoudad-like *M. conjunctivae* (mean: 6.33%, range: 6.12–7.67%). The mean genetic distance among all aoudad individuals (Clade C) included in the study was 0.22% (range: 0.00–1.06%).

**Figure 3:**
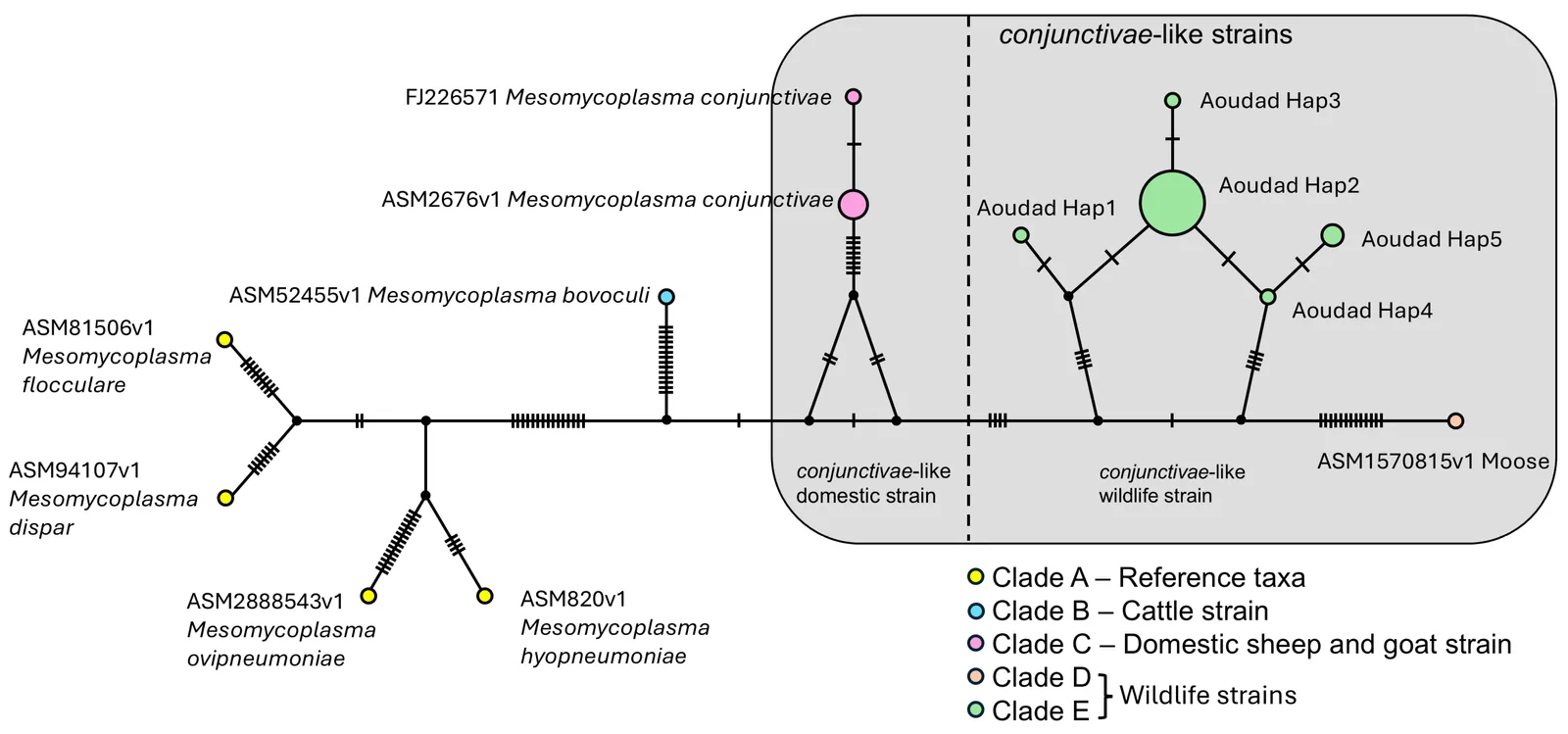
Haplotype network of reference *Mesomycoplasma* species and the 28 16S rRNA sequences of interest from free-ranging aoudad hosts in western Texas.

Six genetic indices were estimated from the 16S rRNA dataset. These included: number of polymorphic sites (*s*) was 75; nucleotide diversity (π) was 0.0465; number of haplotypes (*h*) was 13; haplotype diversity (H_d_) was 0.676; and Fu’s test of neutrality was 4.003; Tajima’s D was −1.48 and was not significant (*p* > 0.10) using DNAsp v6. The 13 haplotypes were visualized using PopART and TCS Networks. Five aoudad-like *M. conjunctivae* haplotypes were identified as follows: Hap 1 includes OR552916 (TK256648); Hap 2 includes OR552904 (TK249443), OR552905 (TK256522), OR552908 (TK256619), OR552911 (TK256637), OR552912 (TK256638), OR552913 (TK256643), OR552914 (TK256644), OR552915 (TK256647), OR552917 (TK256649), OR552918 (TK256654), PZ638880 (TK259803), PZ638882 (TK259806), PZ638883 (TK259807), PZ638884 (TK259808), PZ638885 (TK259809), PZ638886 (TK259811), PZ638887 (TK259812), PZ638888 (TK259813), PZ638890 (TK259815), PZ638891 (TK259816), PZ638892 (TK259818), and PZ638893 (TK259820); Hap 3 includes OR552910 (TK256634); Hap 4 includes PZ638881 (TK259805); and Hap 5 includes PZ638879 (TK259801), PZ638889 (TK259814), and PZ638894 (TK259824) (Figure 3). The ASM2676v1 *Mesomycoplasma conjunctivae* haplotype also includes NR044781, U44770, and NR074135. Overall, the aoudad-like *M. conjunctivae* were separated from each other by one to four nucleotide differences, 18–19 from the moose-like *M. conjunctivae*, and 19–21 from the domestic goat-like *M. conjunctivae* (Figure 3).

#### 3.1.3. Diversity and Haplotype Network for *M. ovipneumoniae*

**Figure 4:**
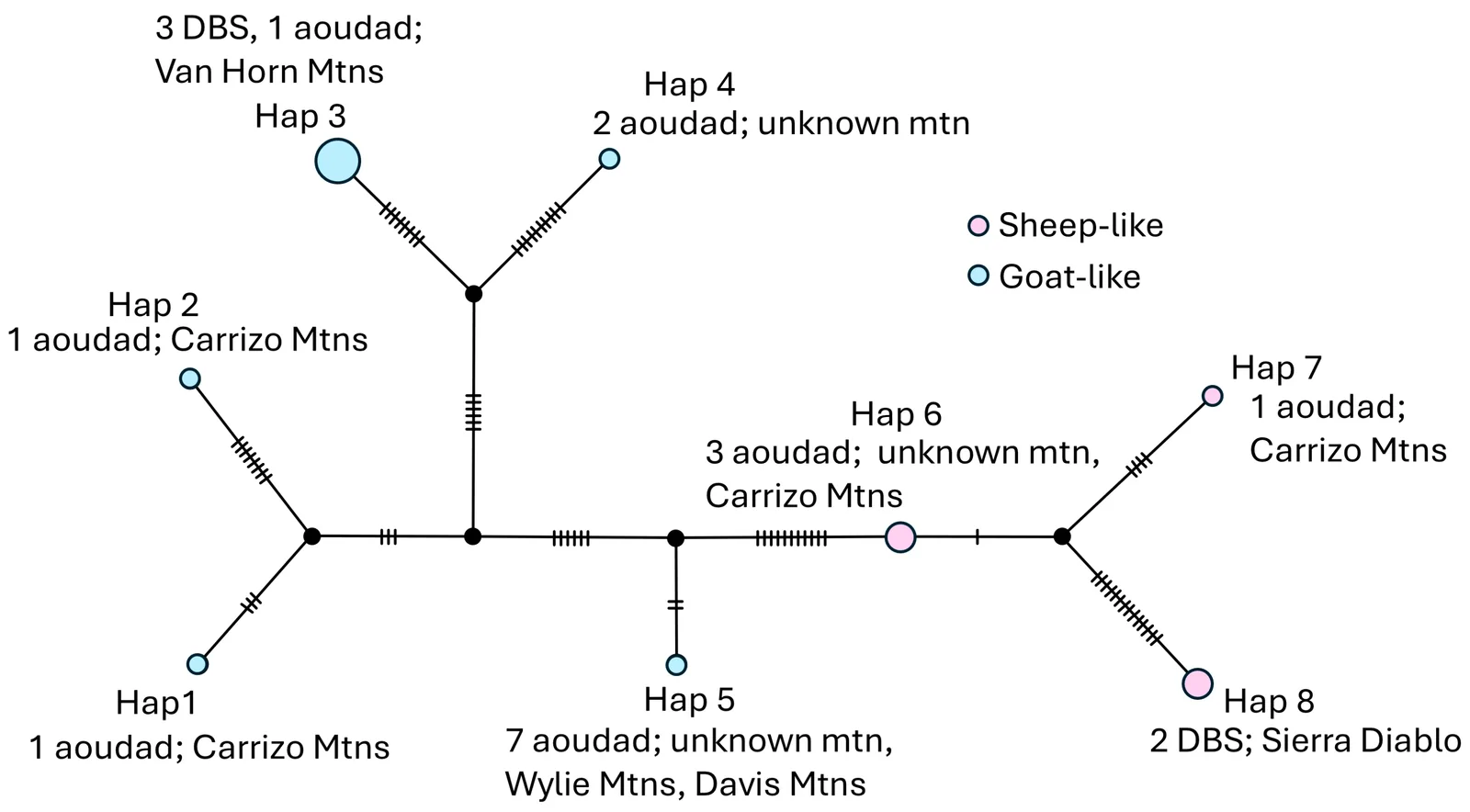
Haplotype network of *rpo*B sequences representative of *Mesomycoplasma ovipneumoniae* from free-ranging aoudad hosts in western Texas. The pink and blue circles represent the sheep-like and goat-like strains of *M. ovipneumoniae*.

Six genetic indices were estimated from the *rpo*B dataset. These included: number of polymorphic sites (*s*) was 45; nucleotide diversity (π) was 0.0306; number of haplotypes (*h*) was 8; haplotype diversity (H_d_) was 0.848; and Fu’s test of neutrality was 6.865; Tajima’s D was 0.8890 and was not significant (*p* > 0.10) using DNAsp v6. The eight haplotypes were visualized using PopART and TCS Networks. Eight haplotypes (three sheep-like and five goat-like strains of *M. ovipneumoniae*) were identified as follows: Hap 1 includes PZ674027 (TK259809); Hap 2 includes PZ674030 (TK259820); Hap 3 includes OR552584 (TK256544), OR552585 (TK256561), OR552586 (TK256575), and OR552587 (TK256601); Hap 4 includes PQ634042 and PQ634043; Hap 5 includes PQ634040, PQ634044, PQ634045, PQ634046, PQ634047, PQ634048, and PQ634049; Hap 6 includes PQ634041, PZ674026 (TK259808), and PZ674029 (TK259818); Hap 7 includes PZ674028 (TK259810); and Hap 8 includes PX505954 and PX505955 (Figure 4). The genetic distances among the eight haplotypes representative of *M. ovipneumoniae* from DBS and aoudad hosts ranged from 0.80 to 4.95% (Table 2).

### 3.2. Statistical and Community Ecology Analyses

#### 3.2.1. Characterization of Nasal and Tonsil Microbiome Diversity

**Figure 5:**
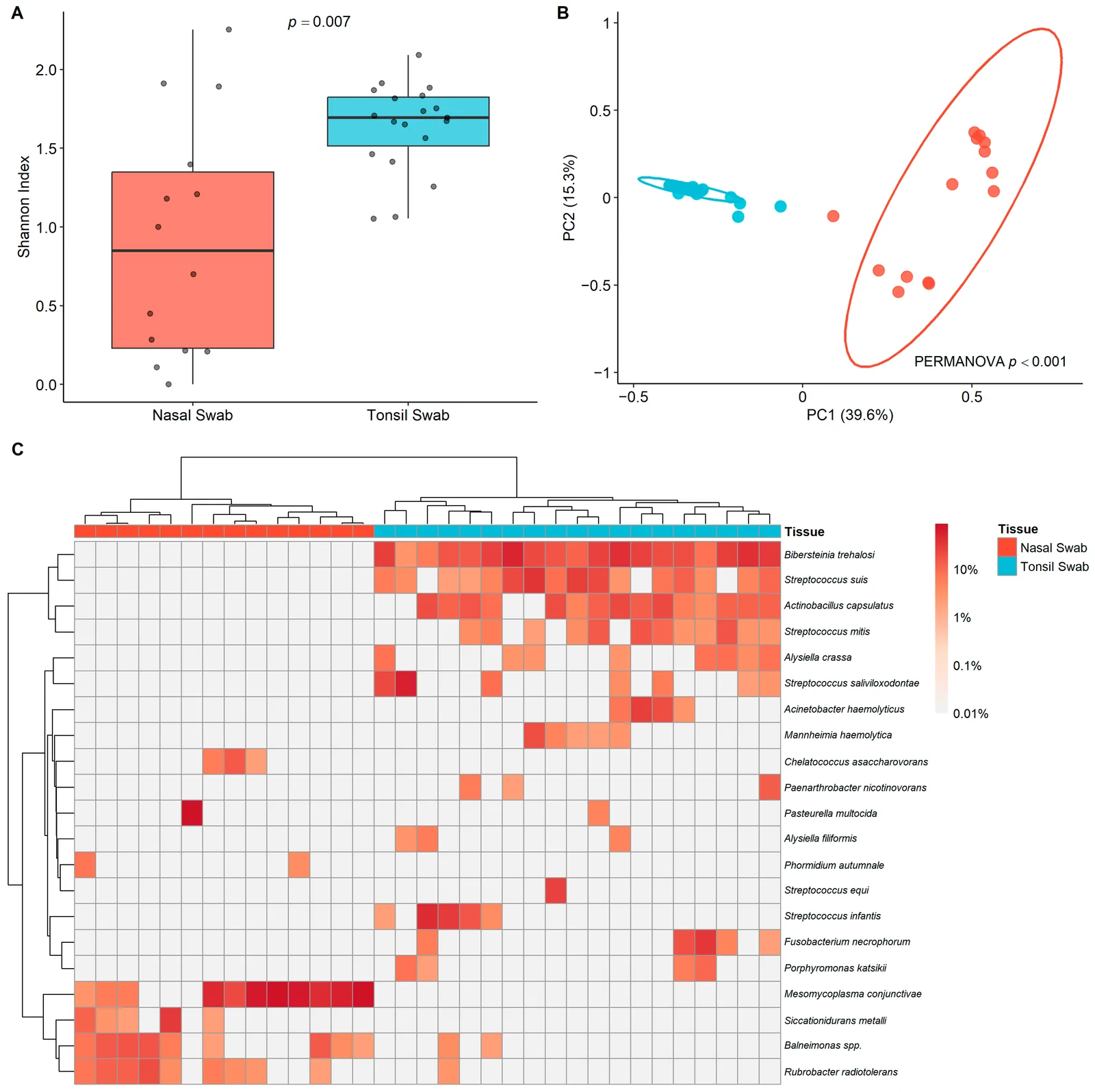
Microbial community diversity, structure, and taxonomic composition across respiratory niches. (**A**) Alpha diversity as measured by the Shannon Diversity Index. The nasal cavity exhibits significantly lower microbial complexity compared to the tonsillar niche (two-sided Wilcoxon Rank-Sum test, *p* = 0.007). Horizontal bars represent medians, boxes outline the interquartile range (IQR), and whiskers extend to 1.5 × IQR. (**B**) Principal Coordinates Analysis (PCoA) ordination based on Bray–Curtis dissimilarities. Clear segregation is observed between nasal (red) and tonsillar (blue) swab profiles, with anatomical niche acting as the primary significant driver of community composition (PERMANOVA, R^2^ = 0.304, *p* = 0.001). Host sex (*p* = 0.156) and age (*p* = 0.487) did not significantly influence clustering. (**C**) Hierarchical clustering heatmap displaying relative abundances of the “hybrid” core microbiome (the 20 most abundant and 20 most discriminatory taxa identified via SIMPER analysis). Column annotations denote the tissue type, host sex, and host age class for each of the 33 processed samples. Abundances are Log_10_transformed (pseudocount = 0.01) and clustered using Ward’s hierarchical clustering method (ward.D2), demonstrating strict clustering by anatomical site rather than host demographic background.

To assess community complexity across respiratory niches (nasal and tonsil cavities), alpha diversity was quantified using the Shannon Diversity Index. A non-parametric comparison revealed that microbial community richness and evenness were significantly lower within the nasal cavity compared to the tonsil cavity (two-sided Wilcoxon Rank-Sum test, W = 55, *p* = 0.007; Figure 5A). This tissue-specific difference was validated in a sub-analysis restricted to the 12 individuals with paired samples (paired Wilcoxon Signed-Rank test, V = 9, *p* = 0.016), which confirmed that host dependency did not artificially drive the observed divergence. Conversely, host demographic variables did not significantly influence community complexity. Shannon Diversity Index values remained statistically uniform across all evaluated age cohorts (Kruskal–Wallis Rank-Sum test, Χ^2^= 2.6443, df = 5, *p* = 0.7546) and demonstrated no significant variance between male and female hosts (Wilcoxon Rank-Sum test, W = 173, *p* = 0.178).

#### 3.2.2. Microbial Community Composition and Niche Differentiation

Principal Coordinates Analysis (PCoA) based on Bray–Curtis distances revealed a clear visual and statistical segregation between the microbial communities inhabiting the nasal and tonsil environments (Figure 5B). To isolate the specific environmental and host factors driving this variation, a multi-factor PERMANOVA evaluated by marginal effects was conducted. The anatomical niche (tissue type, nasal or tonsil swab) was determined to be the primary driver of microbial community structure, independently accounting for 30.4% of the total compositional variance (R^2^ = 0.304, F = 14.96, *p* = 0.001; Table 3). Importantly, these compositional differences were driven by true spatial segregation rather than unequal variance, as multivariate dispersion did not significantly differ between tissue types (permutation test for homogeneity of dispersion, F = 0.417, *p* = 0.528). In contrast, host demographic factors exerted minimal, non-significant effects on the overall community profiles (Figure S1A,B), with host sex explaining 2.9% of the variance (R^2^ = 0.029, *p* = 0.156) and host age explaining 9.9% across the five cohorts (R^2^ = 0.099, *p* = 0.487).

#### 3.2.3. Taxonomic Drivers of Niche Identity

Hierarchical clustering was applied to a hybrid core matrix, comprising the 20 most abundant overall taxa and the 20 most discriminatory taxa, and clearly illustrated the relative abundance patterns that distinguish the nasal and tonsil environments (Figure 5C). Similarity Percentage (SIMPER) analysis identified a small cohort of niche specialists responsible for driving the observed community dissimilarity (Table 4 and Table S3).

The top two operational taxonomic units (OTUs), *M. conjunctivae* and *Bibersteinia trehalosi*, collectively accounted for nearly 40% of the total community variance between the two sites (Table 4). *M. conjunctivae* was identified as the single largest driver of niche differentiation, contributing 23.54% to the total observed dissimilarity and exhibiting massive enrichment within the nasal cavity. Conversely, the tonsil cavity was anchored by a robust complex of Pasteurellaceae and Streptococcaceae, driven primarily by *B. trehalosi* (15.98% contribution), *Actinobacillus capsulatus* (7.03%), and *Streptococcus suis* (6.67%). Minor but distinct nasal specialists included the environmental taxa *Balneimonas* spp. (4.42%) and *Rubrobacter radiotolerans* (3.84%), which further resolved the baseline differences between the two mucosal environments. Together, these top ten distinct operational taxonomic units accounted for 75.9% of the total structural variance between the two mucosal environments.

## 4. Discussion

Nasal and tonsil swabs from 21 aoudad from the Carrizo Mountains, Texas, were used to characterize the nasal and tonsil microbiome relative to identifying causative bacteria associated with IKC. Bioinformatic reports produced by MicroGenDX identified 11 of the 21 aoudad to possess *M. conjunctivae* within the nasal cavity, among other bacterial species. Our data processing methodology demonstrated that all 21 aoudad possessed *M. conjunctivae* in either nasal or tonsil cavities; however, some of these sequences did not possess sufficient coverage to confidently produce accurate 16S rRNA sequence data. For example, the 16S rRNA raw sequence produced by MicroGenDX for TK259803 and TK259820 did not have the coverage to confidently state that there is *M. conjunctivae* in this sample; however, using the 16S rRNA protocol in [14] produced veritable sequence data and therefore was included in all analyses. Subsequently, a total of 16 of the 21 individuals (10 individuals with 16S rRNA sequence data using MicroGenDX and the 16S protocol from [14]; four individuals with only 16S rRNA sequence data using MicroGenDX; two individuals with 16S rRNA sequence data using only the 16S protocol from [14]) from the Carrizo Mountains circa 2020 were confirmed, with high-quality sequence, to possess *M. conjunctivae*.

Sequences from 16 of the 21 aoudad from the Carrizo Mountains were combined with sequences from the 12 aoudad from the Chinati, Sierra Vieja, and Van Horn Mountains previously proposed to have *M. conjunctivae* [14] for a total of 28 *M. conjunctivae* strains from Texas aoudad hosts to compare to *M. conjunctivae* strains detected in domestic goats and a wild moose [26]. Based on phylogenetic analyses, including a neighbor-joining tree (Figure 2), genetic distances (Table 1), and haplotype network (Figure 3), it is apparent that there is a broad distinction between livestock and wild ungulate strains of *M. conjunctivae,* as all goat-like strains were distinct from the aoudad and moose-like strains. The aoudad-like strains differed by two nucleotides and may represent a potential variant or be a result of the sequencing method. Further, Clades C (domestic goat strain), D (wildlife strain-moose), and E (wildlife strain-aoudad) were moderately supported, leaving their association with Clade A unresolved. The apparent lack of support is the result of divergence and genetic dissimilarity between the wildlife strains and the domestic sheep and goat strains. Both strains have similar genetic distance values (Table 1) and are equally likely to be sister to *M. bovoculi*. These unresolved relationships between the bacterial species that cause IKC in cattle, domestic sheep and goats, and wildlife were represented by all three phylogenetic methods (Figure 2 and Figure 3, Table 1).

Interestingly, one sequence (U44770) representative of the goat-like strain herein and a known strain in the IKC epizootic event between domestic goats and desert bighorn in Arizona circa 2004 [20] was identical to the IKC outbreak (causative agent: *M. conjunctivae*) in Norwegian muskox (*Ovibos moschatus*) in 2014 [22]. Both of these epizootic events were most likely caused by domestic goats [20] and sheep [22], which further drives the hypothesis that the wildlife strains of *M. conjunctivae* in both moose and aoudad may be innate to these wild species and not caused by sympatry with domestic livestock.

Although it is beyond the scope of this study, the levels of genetic diversity between the *M. conjunctivae* residing within hosts of moose and aoudad approach the level of diversity between either wildlife haplotype of *M. conjunctivae* and livestock *M. conjunctivae* strains. Further data are required to determine the breadth of diversity among livestock and wildlife strains of *M. conjunctivae* and whether these represent separate variants of *M. conjunctivae* or novel species.

As previously mentioned, these 21 aoudad from the Carrizo Mountains were used for inoculation trials by [23]. In that study, initial *M. ovipneumoniae* strain data were unavailable. Although three aoudad perished prior to the start of inoculations (TK259810: AD#11, TK259818: AD#18, and TK259820: AD#20), one died of haemonchosis (TK259808: AD#9), and one was euthanized 46 days post-inoculation (TK259809: AD#8); it is clear, by the *rpo*B dataset presented herein, that five of the 21 individuals possessed high strain diversity, representing four haplotypes (Figure 4). Two of the identified haplotypes were similar to sheep-like strains of *M. ovipneumoniae,* and the other two haplotypes were representative of goat-like strains of *M. ovipneumoniae*. These individuals possessed at least two species of *Mesomycoplasma* (Figure 2, Figure 3 and Figure 4) as well as several other bacterial species (Figure 5, Table S2).

Upon further examination of the tissue type (nasal and tonsil swabs), a remarkable distinction between these two microbial environments was discovered. The nasal microbiome contains significantly fewer bacterial species than the tonsil microbiome. Although *M. conjunctivae* is a real and potentially detrimental bacterial pathogen, the other bacterial species, *Balneimonas* spp. and *Rubrobacter radiotolerans* (Table 4), detected within the nasal cavity are by-products of the capture method and environment. Because net-gunning from a helicopter usually involves catching the animals at a sprint, most animals tumble and most likely inhale some dirt. Further, subsequent “workup” of animals involved dusty conditions from winds and updrafts caused by helicopter movements. Both of these species are found in the arid desert soil.

In contrast, several bacterial species were found to inhabit the tonsil cavity. The most prevalent bacterium was *Bibersteinia trehalosi* (Table 4), which is implicated in polymicrobial pneumonia in bighorn sheep [34]. Another species, *Mannheimia haemolytica*, involved with respiratory pneumonia disease in bighorn sheep was also detected. Interestingly, *Streptococcus suis*, a bacterial pathogen that can be transmitted from swine to humans, was also detected in the tonsil cavity of aoudad (Table 4). Overall, there appears to be an ecological niche filtering where the nasal cavity is a selective environment (fewer species can survive there), whereas the tonsil cavity permits a wider variety of commensal bacteria (Figure 5, Table 4).

Although this study confirms the presence of a wildlife strain of *M. conjunctivae* in aoudad and describes the microbiome in both nasal and tonsil cavities in select aoudad, there are some notable deficiencies that need to be addressed in future research. First, whole-genome sequencing would allow for complete phylogenetic resolution among *Mesomycoplasma* species and allow for the production of specific primers to distinguish between *M. conjunctivae* and *M. ovipneumoniae* in traditional, quantitative, and digital PCR. Second, samples from Texas aoudad have been limited to only nasal and tonsil swabs, and the lack of samples from the current IKC outbreak limits our ability to associate historical samples with ongoing events. Future sampling events, whether live-captures or opportunistic (e.g., hunting), may need to incorporate swabbing eye lesion sites to evaluate the microbiomes between IKC-affected individuals and asymptomatic individuals.

## 5. Conclusions

Herein, we used an external laboratory (MicroGenDX) to conduct 16S rRNA sequencing on nasal and tonsil swabs from aoudad captured in the Carrizo Mountains, Texas. The combined efforts of the present study and [14] confirmed the existence of an IKC-related pathogen (i.e., the presence of *M. conjunctivae*) that has been circulating in free-ranging aoudad located across four mountain ranges since 2018.

With the current outbreak of IKC circa December 2025 in populations of aoudad in western Texas, it is crucial to identify if the current outbreak is caused by *M. conjunctivae*, as detected herein in previous years, or *Moraxella* species, as proposed by TPWD veterinarians [19]. If it is *M. conjunctivae*, sampling and testing are necessary management efforts to determine whether the strain identified from 2018 to 2020 is capable of causing IKC and went undetected for almost a decade, or a more virulent, novel strain is responsible for the current IKC outbreak. Although IKC has not been documented in DBS populations in Texas to date, direct aoudad management should be considered part of DBS conservation. Knowledge gained from a previous IKC transmission from domestic goats to DBS in Arizona [20] can be used to implement effective medicinal tools (e.g., oxytetracycline) to prevent or reduce spillover into DBS populations. With DBS populations being fairly restricted to sky islands in Texas and given the difficulties of capturing these species in rugged, montane areas, disease surveillance of DBS and sympatric aoudad individuals may only be feasible through boots-on-the-ground fieldwork, camera traps, and potentially drones.

As more research focuses on disease dynamics between native DBS and non-native aoudad [14,15,23,35], sequencing studies are critical to examine the microbiome and to identify bacterial load, composition, and strain type. Although multi-locus sequence typing (MLST) protocols have been the gold standard for identifying pneumonia in bighorn sheep across North America, recent improvements in methodologies (e.g., next-generation sequencing, NGS) allow for a more complete and accurate assignment of sequences to strains. For example, examining only one bacterial species to understand pneumonia in wild sheep is not sufficient to capture the disease dynamics (i.e., sources of infection, transmission pathways, bacterial interactions in respiratory pathways, etc.) and evolutionary histories. With the abundance of research surrounding episodic pneumonia events in bighorn sheep, there remains a need for a robust synthesis of the naturally occurring bacterial species in this ecosystem to explain this conundrum. Consequently, it is time to supplement the MLST toolbox with NGS to better understand the dynamics and interactions among all bacterial species in respiratory microbiomes.

## Figures and Tables

**Table 1:** Genetic distances, shown as percentages, among 16s rRNA sequences representative of *Mesomycoplasma* species estimated using the p-distance model in MEGA12 [29]. *M. conjunctivae* is represented by four identical 16s rRNA sequences and FJ226571. Haplotypes 1–5 are representative of an aoudad-like *M. conjunctivae*. ASM1570815v1 is representative of a moose-like *M. conjunctivae*.

Species Comparison	1	2	3	4	5	6	7	8	9	10	11	12
*flocculare*												
2. *dispar*	4.29											
3. *ovipneumoniae*	7.07	6.31										
4. *hyopneumoniae*	4.56	5.06	5.57									
5. *bovoculi*	12.15	12.15	13.42	11.42								
6. *conjunctivae*	10.27	10.53	13.42	10.62	7.47							
7. FJ226571	10.66	10.41	13.71	10.94	7.89	0.32						
8. Haplotype 1	11.73	11.73	12.05	10.78	9.15	6.19	6.52					
9. Haplotype 2	9.83	9.83	10.05	9.43	7.95	6.12	6.47	0.65				
10. Haplotype 3	10.50	10.50	10.50	9.97	8.31	6.35	6.63	0.97	0.28			
11. Haplotype 4	11.32	11.32	12.58	10.73	8.83	6.74	7.28	1.05	0.33	0.70		
12. Haplotype 5	11.75	11.75	13.02	11.15	9.24	7.13	7.67	1.42	0.68	1.06	0.32	
13. ASM1570815v1	9.37	10.38	11.14	9.39	9.37	7.98	8.40	6.52	5.05	5.53	5.35	5.71

**Table 2:** Genetic distances, shown as percentages, among *rpo*B sequences representative of *Mesomycoplasma ovipneumoniae* estimated using the p-distance model in MEGA12 (Kumar et al. 2024) [29].

Species Comparison	1	2	3	4	5	6	7
Haplotype 1							
2. Haplotype 2	2.24						
3. Haplotype 3	3.95	4.26					
4. Haplotype 4	3.92	4.80	3.38				
5. Haplotype 5	2.49	3.20	4.09	3.92			
6. Haplotype 6	4.10	4.38	4.89	3.92	2.31		
7. Haplotype 7	3.72	3.58	4.95	4.09	3.20	0.80	
8. Haplotype 8	3.08	3.62	4.61	4.16	3.44	2.71	3.26

**Table 3:** Multi-factor PERMANOVA partitioning the marginal effects of tissue type, sex, and age class on microbial community dissimilarity (Bray–Curtis distance).

Variable	Df	Sum of Squares	R^2^	F	Pr(>F)
Tissue type	1	3.5548	0.3039	14.9571	0.001
Sex	1	0.3440	0.0294	1.4474	0.156
Age class	5	1.1569	0.0989	0.9736	0.487
Residuals	25	5.9416	0.5080		
Total	32	11.6956	1.0000		

**Table 4:** Top 10 taxonomic contributors to the microbial community dissimilarity between nasal and tonsillar niches as identified by SIMPER analysis.

Taxon	Primary Niche	Dissimilarity Contribution (%)	Cumulative Contribution (%)
*Mesomycoplasma conjunctivae*	Nasal	23.54%	23.54%
*Bibersteinia trehalosi*	Tonsil	15.98%	39.52%
*Actinobacillus capsulatus*	Tonsil	7.03%	46.55%
*Streptococcus suis*	Tonsil	6.67%	53.22%
*Balneimonas* spp.	Nasal	4.42%	57.64%
*Streptococcus saliviloxodontae*	Tonsil	3.88%	61.52%
*Rubrobacter radiotolerans*	Nasal	3.84%	65.36%
*Pasteurella multocida*	Tonsil	3.80%	69.16%
*Streptococcus infantis*	Tonsil	3.56%	72.72%
*Streptococcus mitis*	Tonsil	3.14%	75.86%

## Data Availability

16S rRNA and *rpo*B sequences are available at NCBI GenBank with accession numbers PZ638879-PZ638894 and PZ674026-PZ674030, respectively. Raw 16S rRNA data files were accessioned under PRJNA1498207 in the NCBI BioProject database with BioSample numbers SAMN61833159-SAMN1833179 and SRR39752494-SRR39752481 in the NCBI SRA database. Code for statistical analyses is available at https://github.com/emilwrig/aoudad_M_conjunctivae accessed on 19 August 2026 and archived at Zenodo (https://zenodo.org/records/21464767 accessed on 19 August 2026; 10.5281/zenodo.21464767).

## Supplementary Materials

The following supporting information can be downloaded at: https://www.mdpi.com/article/10.3390/ani16172639/s1, Figure S1: Non-significant demographic interactions by host age and sex; Table S1: Specimen examined; Table S2: Bacterial incidence; Table S3: SIMPER results.

## Abbreviations

The following abbreviations are used in this manuscript:

DBS	Desert bighorn sheep
IKC	Infectious keratoconjunctivitis
IQR	Interquartile range
MLST	Multi-locus sequence typing
OTU	Operational taxonomic unit
PERMANOVA	Permutational multivariate analysis of variance
PCoA	Principal coordinates analysis
SIMPER	Similarity percentage
TPWD	Texas Parks and Wildlife Department

## References

[1] McCabe R.E., McCabe T.R., Halls L.K. (1984). Of Slings and Arrows: An Historical Retrospection. White-Tailed Deer: Ecology and Management.

[2] Bean W.T., Butterfield H.S., Fiehler C., Hacker D., Howard J.K., Namitz R., Swanson B., Batter T.J. (2024). Contrasting Management Paradigms for Pronghorn in the Arid Southwest and Their Northern Range: A Review. J. Wildl. Manag..

[3] Stroupe S., Forgacs D., Harris A., Derr J.N., Davis B.W. (2022). Genomic Evaluation of Hybridization in Historic and Modern North American Bison (*Bison bison*). Sci. Rep..

[4] Whiting J.C., Bleich V.C., Bowyer R.T., Epps C.W. (2023). Restoration of Bighorn Sheep: History, Successes, and Remaining Conservation Issues. Front. Ecol. Evol..

[5] Davis W.B., Taylor W.P. (1939). The Bighorn Sheep of Texas. J. Mammal..

[6] Cook R.L. (1992). A Historical Review of Reports, Field Notes, and Correspondence on the Desert Bighorn Sheep in Texas: Special Report to the Desert Bighorn Sheep Advisory Committee: Contribution of Federal Aid Project W-127-R and W-123-D.

[7] Kilpatric J. (1982). Status of Desert Bighorn Sheep in Texas—1982. Desert Bighorn Counc. Trans..

[8] Đurić M. (2022). Orbivirus caerulinguae (Bluetongue Virus).

[9] Wright E.A., Wiedmeier R.C., Roberts E.K., Pipkin D.R., Hernández F., Bayouth J.P., Conway W.C., Bradley R.D. (2022). Distinct MtDNA Lineages in Free-ranging *Ammotragus* (Aoudad) from the United States Indicate Multiple Introductions from Northern Africa. Ecol. Evol..

[10] Wright E.A., Bradley R.D., Manthey J.D. (2024). Translocations, Rising Populations, and Phylogeographic Consequences: Genomic Implications for Conservation of Introduced Aoudad (*Ammotragus lervia*) in the Southwestern United States. J. Mammal..

[11] Wright E.A., Bradley R.D. (2023). Genetic Divergence and MtDNA Lineages in Six Subspecies of Aoudad.

[12] Weidmeier R.C. (2021). Characterization of Aoudad and Desert Bighorn Sheep Microbiomes in Association to Disease Risk.

[13] Wilcox D.C. (2022). Interactions between Aoudad, Desert Bighorn Sheep, and Mule Deer in the Texas Trans-Pecos.

[14] Wright E.A., Brugette G.G., Buckert K.F., Hernández F., Reed J.H., Wyckoff S.R., Taylor J.C., Manlove K.R., Phillips C.D., Bradley R.D. (2024). Multi-locus Sequence Typing Indicates Multiple Strains of *Mycoplasma* in Desert Bighorn Sheep and Aoudad in Texas. J. Wildl. Manag..

[15] Thomas L.F., Clontz D., Nunez C.M., Richison J.J., Dittmar R.O., Reed J.H., Wyckoff S.R., Hernandez F., Gray S.S., Rech R.R. (2026). Aoudad (*Ammotragus lervia*) as Disease Reservoirs for Bighorn Sheep (*Ovis canadensis*): Experimental and Epidemiological Evidence. Sci. Rep..

[16] Traweek M., Welch R. (1992). Exotics in Texas.

[17] Saldaña L. (2025). Back from the Brink-Again? Texas Landowners and Wildlife Experts Confront Latest Bighorn Crisis. Tex. Wildl..

[18] Neha Sufia A., Wright E.A., Brugette G.G., Buckert K.F., Phillips C.D., Bradley R.D. (2026). Metagenomic Analysis of Diets in Sympatric Aoudad, Desert Bighorn Sheep, and Mule Deer in the Chihuahuan Desert. Ecol. Evol..

[19] Texas Parks and Wildlife Department (2026). Status Update.

[20] Jansen B.D., Heffelfinger J.R., Noon T.H., Krausman P.R., Devos J.C. (2006). Infectious Keratoconjunctivitis in Bighorn Sheep, Silver Bell Mountains, Arizona, USA. J. Wildl. Dis..

[21] Jansen B.D., Krausman P.R., Heffelfinger J.R., Noon T.H., Devos J.C. (2007). Population Dynamics and Behavior of Bighorn Sheep with Infectious Keratoconjunctivitis. J. Wildl. Manag..

[22] Handeland K., Madslien K., Bretten T., Røtvei I., Våge J., Tengs T. (2020). Mycoplasma Conjunctivae-Associated Keratoconjunctivitis in Norwegian Muskox (*Ovibos moschatus*). J. Wildl. Dis..

[23] Thomas L.F., Clontz D., Nunez C.M., Dittmar R.O., Hernandez F., Rech R.R., Cook W.E. (2024). Evaluating the Transmission Dynamics and Host Competency of Aoudad (*Ammotragus lervia*) Experimentally Infected with Mycoplasma Ovipneumoniae and Leukotoxigenic Pasteurellaceae. PLoS ONE.

[24] Crawford E.D., Martin R., Phillips C.D., Stanton W.N., van Bokhoven A., Lucia M.S., Arangua P.B., La Rosa F.G., Grasmick Z., Terlecki R. (2023). Microbiomes in Post–Digital Rectal Exam Urine Samples Are Linked to Prostate Cancer Risk. JU Open Plus.

[25] Robinson J.T., Thorvaldsdóttir H., Winckler W., Guttman M., Lander E.S., Getz G., Mesirov J.P. (2011). Integrative Genomics Viewer. Nat. Biotechnol..

[26] Herndon D.R., Beckmen K.B., Highland M.A. (2021). Draft Genome Sequence of a Novel Mycoplasma Species Identified from the Respiratory Tract of an Alaska Moose (*Alces alces gigas*). Microbiol. Resour. Announc..

[27] Swofford D.L. (2003). PAUP*: Phylogenetic Analysis Using Parsimony (* and Other Methods).

[28] Felsenstein J. (1985). Confidence Limits on Phylogenies: An Approach Using the Bootstrap. Evolution.

[29] Kumar S., Stecher G., Suleski M., Sanderford M., Sharma S., Tamura K. (2024). MEGA12: Molecular Evolutionary Genetic Analysis Version 12 for Adaptive and Green Computing. Mol. Biol. Evol..

[30] Rozas J., Ferrer-Mata A., Sánchez-DelBarrio J.C., Guirao-Rico S., Librado P., Ramos-Onsins S.E., Sánchez-Gracia A. (2017). DnaSP 6: DNA Sequence Polymorphism Analysis of Large Data Sets. Mol. Biol. Evol..

[31] Leigh J.W., Bryant D., Nakagawa S. (2015). POPART: Full-Feature Software for Haplotype Network Construction. Methods Ecol. Evol..

[32] Clement M., Snell Q., Walker P., Posada D., Crandall K. (2002). TCS: Estimating Gene Genealogies. Proceedings of the 16th International Parallel and Distributed Processing Symposium.

[33] Kamath P.L., Manlove K., Cassirer E.F., Cross P.C., Besser T.E. (2019). Genetic Structure of Mycoplasma Ovipneumoniae Informs Pathogen Spillover Dynamics between Domestic and Wild Caprinae in the Western United States. Sci. Rep..

[34] Besser T.E., Cassirer E.F., Highland M.A., Wolff P., Justice-Allen A., Mansfield K., Davis M.A., Foreyt W. (2013). Bighorn Sheep Pneumonia: Sorting out the Cause of a Polymicrobial Disease. Prev. Vet. Med..

[35] Thomas L.F., Panaretos C., Scott M.A., Valeris-Chacin R., Cook W.E. (2026). Distinct Host–Pathogen and Microbiome Responses of Aoudad (*Ammotragus lervia*) and Bighorn Sheep (*Ovis canadensis*) Following Exposure to *Mycoplasma ovipneumoniae* with or without Co-Exposure to Leukotoxigenic Pasteurellaceae. BMC Vet. Res..

