# Supplementary material for: Independent Discovery of Mesomycoplasma conjunctivae in Non-Native Aoudad (Ammotragus lervia) in Texas
Source: Animals (Basel). 2026 Aug 23;16(17):2639. doi: 10.3390/ani16172639 (PMC13564293; doi:10.3390/ani16172639)

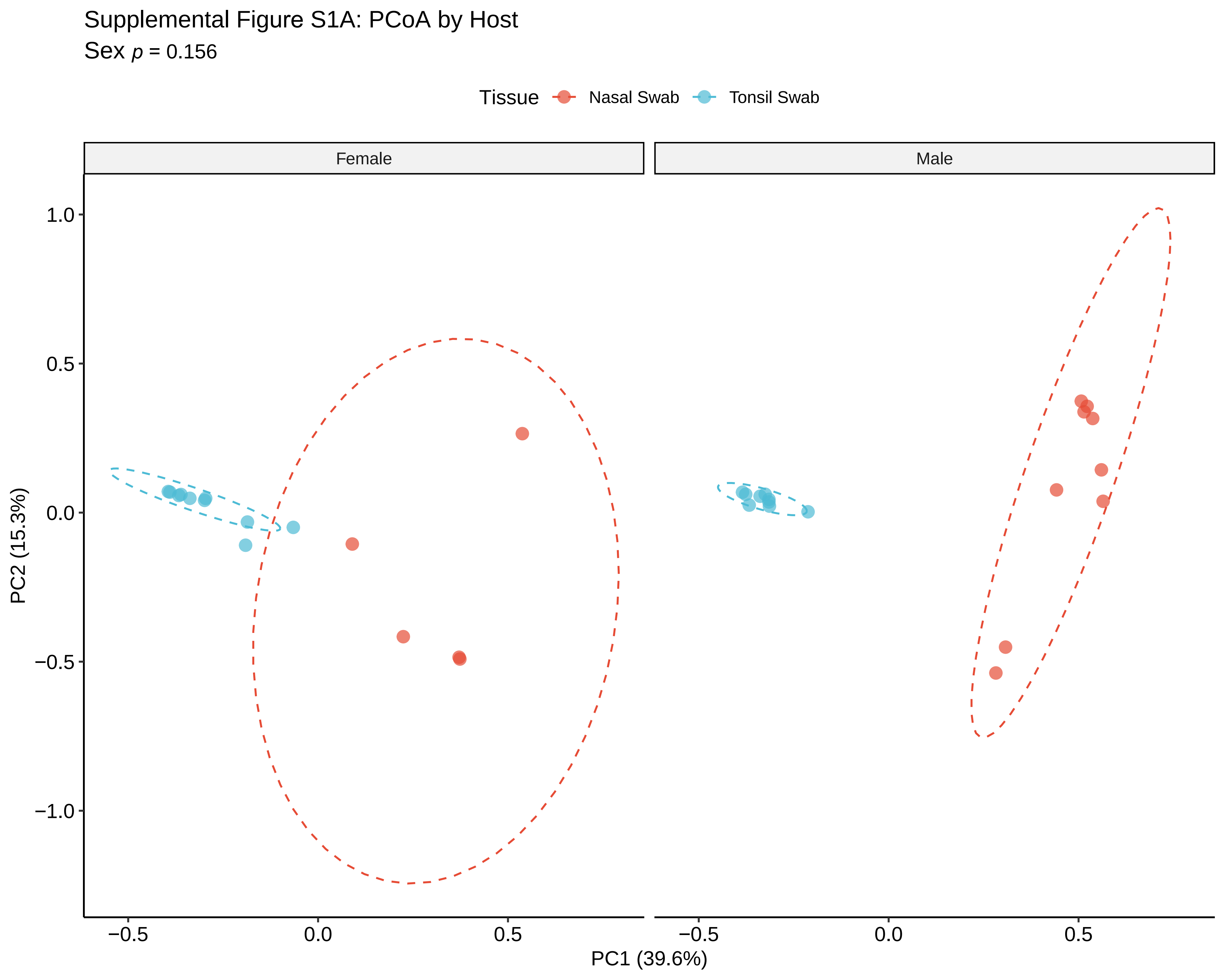

Supplemental Figure S1A: PCoA by Host
Sex p = 0.156
Tissue
Nasal Swab
Tonsil Swab
Female
Male
1.0
0.5
0.0
−0.5
−1.0
PC2 (15.3%)
−0.5
0.0
0.5
PC1 (39.6%)

Supplemental Figure S1B: PCoA by Host

Age p = 0.487

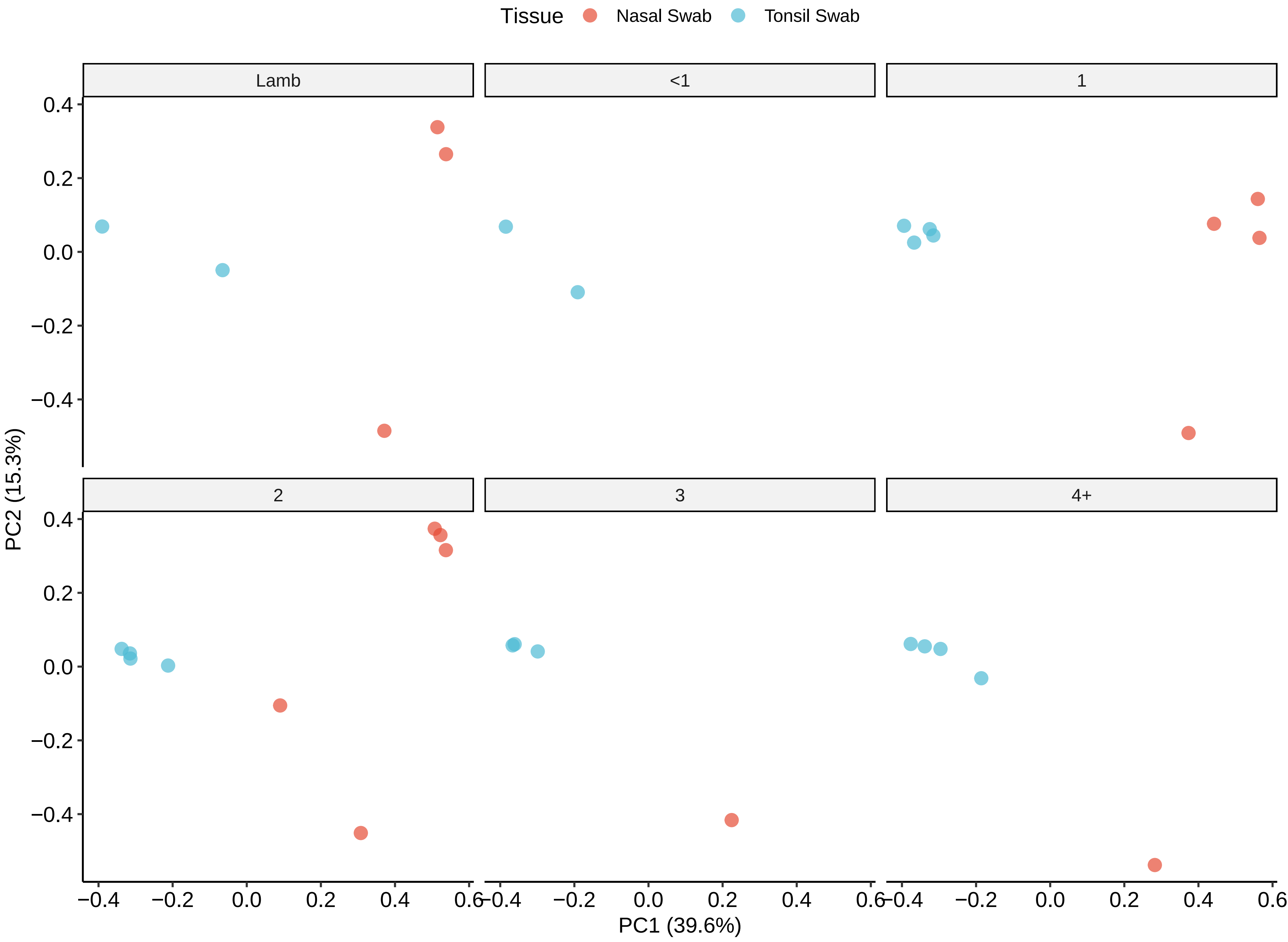

Supplement: Supplementary file 1 [file animals-16-02639-s001.zip › animals-4481642-FigureS1.pdf]
